# Private queries on encrypted genomic data

**DOI:** 10.1186/s12920-017-0276-z

**Published:** 2017-07-26

**Authors:** Gizem S. Çetin, Hao Chen, Kim Laine, Kristin Lauter, Peter Rindal, Yuhou Xia

**Affiliations:** 1 Worcester Polytechnic Institute, 100 Institute Rd, Worcester, MA 01609 USA; 2 Microsoft Research, 14820 NE 36th St, Redmond, WA 98052 USA; 3 Oregon State University, 2500 NW Monroe Ave, Corvallis, OR 97331 USA; 4 Princeton University, 304 Washington Rd, Princeton, NJ 08544 USA

**Keywords:** Cryptography, Homomorphic encryption, Genome privacy

## Abstract

**Background:**

One of the tasks in the iDASH Secure Genome Analysis Competition in 2016 was to demonstrate the feasibility of privacy-preserving queries on homomorphically encrypted genomic data. More precisely, given a list of up to 100,000 mutations, the task was to encrypt the data using homomorphic encryption in a way that allows it to be stored securely in the cloud, and enables the data owner to query the dataset for the presence of specific mutations, without revealing any information about the dataset or the queries to the cloud.

**Methods:**

We devise a novel string matching protocol to enable privacy-preserving queries on homomorphically encrypted data. Our protocol combines state-of-the-art techniques from homomorphic encryption and private set intersection protocols to minimize the computational and communication cost.

**Results:**

We implemented our protocol using the homomorphic encryption library SEAL v2.1, and applied it to obtain an efficient solution to the iDASH competition task. For example, using 8 threads, our protocol achieves a running time of only 4 s, and a communication cost of 2 MB, when querying for the presence of 5 mutations from an encrypted dataset of 100,000 mutations.

**Conclusions:**

We demonstrate that homomorphic encryption can be used to enable an efficient privacy-preserving mechanism for querying the presence of particular mutations in realistic size datasets. Beyond its applications to genomics, our protocol can just as well be applied to any kind of data, and is therefore of independent interest to the homomorphic encryption community.

## Background

In 2015 and 2016, *iDASH (integrating Data for Analysis, Anonymization, and Sharing)* hosted two international contests on *Secure Genome Analysis*. Teams from around the world participated to test the limits of secure computation on genomic data, and benchmark solutions on real data sets. Such contests serve to bring together experts in security, cryptography, and bioinformatics to quickly make progress on interdisciplinary challenges. The task for outsourced storage and computation this year was to implement a method for private queries—specifically string matching on encrypted genomic data.

### Motivation

In recent years cloud storage and services have been developing rapidly. Enterprise customers in the medical and financial sectors can potentially save money and streamline business processes by outsourcing the storage and computation of their data to public storage clouds. Instead of storing and managing a large amount of data locally, a medical center can utilize cloud storage for electronic medical records or genomic data of patients. However, using public storage clouds can potentially compromise the privacy and security of the data. One effective way of addressing these concerns is to store private data in encrypted form in the public cloud. Typical block ciphers do not allow data to be used in encrypted form, and meaningful computation on the data would either require it to be returned to the customer for decryption, or alternatively for the storage cloud to have access to the decryption key. A new way to solve this problem is to instead encrypt it using a very special encryption scheme that is specifically designed to allow for computations to be done in encrypted form. Such an encryption scheme is called a *homomorphic encryption scheme*.

In this paper we focus on tasks related to *string matching*, which is motivated by the following scenario. A medical center wants to outsource the storage of several VCF files (The Variant Call Format Version 4.2) containing patients’ genomic data to a public cloud. In order to protect the privacy of the patients, the medical center uploads the files in homomorphically encrypted form. At a later date, the medical center needs to calculate the probability of certain genetic diseases through matching a set of biomarkers to the encrypted genomes of patients. These query biomarkers also need to be passed to the cloud in homomorphically encrypted form to protect the privacy of the patients. The cloud then needs to match the biomarkers in the encrypted query to those in the encrypted VCF file using a string matching algorithm compatible with homomorphic encryption. This produces an encrypted result, which it can then send back to the medical center for decryption and analysis.

2In its most basic form, string matching on homomorphically encrypted data can be summarized as follows. Suppose a string *Q* of length *ℓ* has been homomorphically encrypted. Now consider another string *X* of the same length *ℓ*. The task is to perform a comparison operation that returns a ciphertext whose decryption reveals whether *Q* and *X* are the same string. The decryption may or may not leak information about one or both of the strings *Q*, *X*. As a more complicated example, consider a dataset of *N* homomorphically encrypted strings *Q*_1_,…,*Q*_*N*_, all of length *ℓ*. Now the task might be to query the entire dataset, and to return a ciphertext whose decryption reveals whether a given query string *X* is present in the set. Again, the decryption may or may not reveal information about one or more of the strings *Q*_*i*_, *X*. In more complicated examples, one might want to accept partial matches, or strings within a certain Hamming distance of the query string.

### Summary of results

We start by discussing the basics of homomorphic encryption, and techniques for encoding and batching ciphertexts. Next, we outline various approaches for string matching on homomorphically encrypted data. We then employ hashing techniques from Private Set Intersection protocols [1, 2] to obtain significant performance improvements over the basic methods. We then focus on one particular method for string matching, and describe formally a practical and efficient protocol for using it for privacy-preserving queries.

We apply our protocol to solve the homomorphic encryption challenge in the 2016 iDASH Secure Genome Analysis Competition. In this task, we are given a VCF file containing a certain number of rows (each row corresponds to a mutation). From each row we extract 40 bits of relevant information, encrypt it using a homomorphic encryption scheme, and upload it to a cloud server. A client queries the server for the presence of a particular mutation (single query) or several mutations (multiquery) in the file. The server performs the query matching homomorphically, and returns the encrypted result to the client. For homomorphic encryption, our solution uses the Fan-Vercauteren scheme from [3], and the implementation in the *Simple Encrypted Arithmetic Library - SEAL* [4]. We evaluated our solution on two example VCF files, containing 10,000 rows and 100,000 rows, respectively. Table 5 summarizes the performance of our solution.

Our solution to the iDASH competition task was selected as the winner at an associated workshop held in Chicago, IL in November 2016.

### Related work

Over the last 5 years, there have been a number of papers demonstrating computation on homomorphically encrypted medical and genomic data, in a line of work starting with [5], which showed how practical statistical tasks can be achieved by introducing a different way of encoding data as polynomials to avoid deep circuits for simple multiplication of real numbers. Other computational tasks that have been demonstrated to be viable on homomorphically encrypted data include heart attack risk prediction from encrypted health data [6], and genomic computations such as Pearson Goodness-of-Fit test, Cochran-Armitage Test for Trend, measures of linkage disequilibrium, and Estimation Maximization algorithm for haplotyping [7]. Homomorphic computation of edit distance was one of the tasks for the 2015 iDASH competition, and was demonstrated to be possible by a number of submissions [8]. A proceedings volume covering the submissions to the 2015 competition was published, including a paper covering one of the winning submissions: an optimized implementation of the modified edit distance algorithm [9, 10]. Several other works have focused on applications of homomorphic encryption on evaluating machine learning models on encrypted data [11, 12].

In Bedö et al. [13] show how to perform very advanced string matching tasks—e.g. fuzzy matching—on homomorphically encrypted data. Unfortunately, these techniques come with a large computational and communication overhead, making them significantly less practical than what we present here.

The homomorphic encryption library SEAL is described in [4, 14].

## Methods

### Homomorphic encryption

Homomorphic encryption is a powerful cryptographic primitive that allows computation to be performed on encrypted data. While the idea in principle is old [15], and many well-known public-key cryptosystems (e.g. RSA, ElGamal, Paillier) already allow either additions or multiplications to be performed on the ciphertext side, only very recently Craig Gentry described the first *fully homomorphic encryption scheme* in the seminal paper [16], where the encryption scheme respects both addition and multiplication. Since then, a vast amount of both theoretical and practical implementation work has been done to improve the efficiency of homomorphic encryption schemes [3, 17–21], and at this point researchers are starting to see performance results that are good enough for carefully selected realistic applications [5, 12, 22].

While many of the techniques and algorithms presented in this paper are agnostic to the exact homomorphic encryption scheme that is being used, for simplicity we will restrict to *Ring LWE*-based cryptosystems using power-of-2 cyclotomic rings of integers [23]. In such cryptosystems, the plaintext space is typically the polynomial quotient ring $\mathbb {Z}_{t}[x]/(x^{n}+1)$, and the ciphertext space the polynomial quotient ring $\mathbb {Z}_{q}[x]/(x^{n}+1)$, where *n* is a power of 2, and *t*≪*q* are positive integers. Here $\mathbb {Z}_{t}$ and $\mathbb {Z}_{q}$ denote integers modulo *t* and *q*, respectively. Thus, it is customary to denote $R = \mathbb {Z}[x]/(x^{n}+1)$, so that the plaintext and ciphertext spaces become *R*_*t*_=*R*/*t* *R*, and *R*_*q*_=*R*/*q* *R*, respectively. We use this notation throughout the paper. We note that in some schemes, such as the BGV scheme described in [19], the modulus *q* changes throughout the homomorphic evaluation as a result of an operation called *modulus switching*, but this fact has no consequences for our protocol, and the reader can safely ignore it. In our experiments we use the Simple Encrypted Arithmetic Library - SEAL [4], which implements the Fan-Vercauteren (FV) scheme described in [3]. Thus, our notation and terminology most closely follow [4] and [3], but the techniques apply trivially also to many other schemes.

#### Leveled fully homomorphic encryption

Technically speaking, *fully* homomorphic encryption refers to an encryption scheme which can evaluate any arithmetic circuit on encrypted inputs. In practice, this turns out to be too much to ask. Instead, by restricting the multiplicative depth of the circuits to some bound *L*, one can set the parameters of the encryption scheme to support only circuits up to depth *L*, and obtain significantly better performance than what a true fully homomorphic encryption scheme would give. Such schemes are called *leveled* fully homomorphic, and are typically described by the following randomized algorithms:

- Setup(1^*κ*^,1^*L*^): Given a security parameter *κ* and a parameter $L \in \mathbb {Z}^{+}$ (level), outputs a set of encryption parameters parms.
- KeyGen(parms): Outputs a secret key sk and a public key pk. Optionally outputs one or more evaluation keys evk.
- Encrypt(*m*,pk): Given message *m*∈*R*_*t*_, outputs ciphertext *c*∈*R*_*q*_.
- Decrypt(*c*,sk): Given ciphertext *c*∈*R*_*q*_, outputs message *m*∈*R*_*t*_.
- Evaluate(*C*,(*c*_1_,…,*c*_*k*_),evk): Given circuit *f* of depth at most *L* with *k* input wires, and inputs *c*_1_,…,*c*_*k*_ with *c*_*i*_→Encrypt(*m*_*i*_,pk), outputs a ciphertext *c* such that
  $$ \text{Pr}\left[ \texttt{Decrypt}(c, \texttt{sk}) \neq f(m_{1},\ldots, m_{k}) \right] = \text{negl}(\kappa) \,. $$
  Moreover, we require that the size of the output of Evaluate is not more than polynomial in *κ* independent of *f* (compactness), and independent of *L* (see e.g. [24]).

We say that a leveled fully homomorphic encryption scheme is secure if it is IND-CPA secure. The reader is referred to [19] for more details.

#### Encoding

As we explained above, we restrict to homomorphic encryption schemes where the plaintext space is the polynomial quotient ring *R*_*t*_. Thus, when integers are to be encrypted, and integer arithmetic performed on them in encrypted form, one needs to employ an *encoding scheme* to convert integers into elements of *R*_*t*_. There are many ways to do this (see e.g. [4]), but we only need the simplest method in this work. Namely, given an integer $m\in \mathbb {Z}$, we encode it as the constant polynomial *m*∈*R*_*t*_. Of course, this allows us to only encode integers between 0 and *t*−1, which gives a strict lower bound on the size of *t* that we can use. The corresponding decoding function is equally trivial: interpret the constant polynomial as an integer. As $\mathbb {Z}_{t}$ is a subring of *R*_*t*_, as long as we ensure that the underlying plaintext integers that are encountered during the homomorphic evaluation never get reduced modulo *t*, we can use the homomorphic encryption scheme to perform integer arithmetic. In practice, this places a strong lower bound on the size of *t*, which subsequently necessitates the use of larger *n* and *q* for technical reasons [4]. Unfortunately, increasing *n* and *q* can have a dramatic adverse effect on performance, which is why it is crucial to choose *t* as small as possible. This typically requires a detailed analysis of the computation that is to be performed on encrypted data.

#### Batching

Batching is a powerful technique that allows SIMD (Single Instruction, Multiple Data) operations to be performed on homomorphically encrypted data. We give a very brief explanation here, and refer the reader to [4, 22, 25, 26] for more details.

The simple encoding scheme described above is extremely wasteful, as it encodes only one single integer modulo *t* into a plaintext polynomial with enough space to store thousands of such integers. Indeed, recall that we use the ring $R=\mathbb {Z}[x]/(x^{n}+1)$ to construct both the plaintext space (*R*_*t*_) and the ciphertext space (*R*_*q*_), and that *n* is always a power of 2. Typically *n* is at least 1024, and in some extreme examples as large as 65536. The size of *q* depends on *n*, and ranges between 30 bits for *n*=1024 to thousands of bits for *n*=65536. A naive way to try to improve the situation by enabling SIMD operations would be to encode one integer modulo *t* into each coefficient of the message polynomial. While such an encoding would indeed work when the additive homomorphism is used (addition of polynomials in *R*_*t*_ is done coefficient-wise), it would not work for multiplications. Instead, the standard approach is to choose *t* such that the polynomial modulus *x* ^*n*^+1 factors into *n* linear factors modulo *t*. This is achieved by restricting *t* to be a prime such that 2*n*|(*t*−1). This causes the plaintext space *R*_*t*_ to split into a direct product as $R_{t}\cong \mathbb {Z}_{t}^{n}$, where the isomorphism is an isomorphism of rings, meaning it respects both additions and multiplications. Given a vector $\vec {m} \in \mathbb {Z}_{t}^{n}$ representing the values in the individual *slots*, we denote its *composition* into a plaintext polynomial *m*∈*R*_*t*_ by $\texttt {Compose}(\vec {m})$. Similarly, given a plaintext polynomial *m*∈*R*_*t*_, we denote its *decomposition* into a vector $\vec {m}\in \mathbb {Z}_{t}^{n}$ representing the values in the individual slots by Decompose(*m*). An explicit description of the isomorphism is given in the references mentioned above.

In computations where SIMD operations can be used, batching can provide an enormous improvement in *latency*, and in other cases at least in *throughput* (see e.g. [12, 22, 25, 26]), making it one of the most powerful and important concepts in homomorphic encryption.

### String matching

#### Setup

Suppose we are given a dataset $\mathcal {D}$ of distinct *N* *ℓ*-bit strings *Q* ^(1)^,…,*Q* ^(*N*)^. We denote the bits in *Q* ^(*i*)^ by $Q^{(i)}_{1}, \ldots, Q^{(i)}_{\ell }$, so the dataset can be organized into an *N*×*ℓ* matrix of bits:

$$\mathcal{D} = \left(\begin{array}{ccc} Q^{(1)}_{1} & \cdots & Q^{(1)}_{\ell} \\ \vdots & \ddots & \vdots \\ Q^{(N)}_{1} & \cdots & Q^{(N)}_{\ell} \end{array}\right) $$

We need to homomorphically encrypt $\mathcal {D}$ to produce an encrypted dataset $\mathcal {D}_{\text {Enc}}$, and we explain below how this is done. Likewise, given an *ℓ*-bit query string *X*, we denote its bits by *X*_1_,…*X*_*ℓ*_, and explain below how to homomorphically encrypt it to yield an encrypted query *X*_Enc_. The task is then to construct a low-depth arithmetic circuit *f*_Query_, such that the result of the homomorphic evaluation $f_{\text {Query}}(\mathcal {D}_{\text {Enc}}, X_{\text {Enc}})$ decrypts and decodes correctly to yield a plaintext from which one can determine whether *X* matches any of the rows of $\mathcal {D}$.

#### Encrypting $\mathcal {D}$ and *X*

To encrypt the dataset, we use homomorphic encryption with batching. Suppose *n* is a power of two, and *t* is a prime such that 2*n*|(*t*−1), so that every plaintext polynomial in *R*_*t*_ can be considered as an *n*-tuple of slots, each containing an integer modulo *t*.

If *n*∤*N*, one can always add empty rows to extend the dataset to satisfy *n*|*N*. Thus, without loss of generality, we will assume *n*|*N*. Let *B*=*N*/*n* be the *batch count*. We form a *B*×*ℓ* matrix $\overline {\mathcal {D}}$ of plaintext polynomials

$${} {{\overline{\mathcal{D}} =\! \left(\begin{array}{ccccc} \texttt{Compose} \left[\begin{array}{c} Q^{(1)}_{1} \\ \vdots \\ Q^{(n)}_{1} \end{array}\right] & & \cdots & & \texttt{Compose} \left[\begin{array}{c} Q^{(1)}_{\ell} \\ \vdots \\ Q^{(n)}_{\ell} \end{array}\right]\\ \vdots & & \ddots & & \vdots \\ \texttt{Compose} \left[\begin{array}{c} Q^{(N-n + 1)}_{1} \\ \vdots \\ Q^{(N)}_{1} \end{array}\right] & & \cdots & & \texttt{Compose} \left[\begin{array}{cl} Q^{(N-n+1)}_{\ell} \\ \vdots \\ Q^{(N)}_{\ell} \end{array}\right] \end{array} \,\right)\!,}} $$

which we encrypt entry-wise to form an encrypted matrix $\overline {\mathcal {D}}_{\text {Enc}}$, whose *i*-th row $\overline {\mathcal {D}}_{\text {Enc}}^{(i)}$ is a vector of length *ℓ* of ciphertext polynomials.

Given a query string *X* with bits *X*_1_,…,*X*_*ℓ*_, we first form an *ℓ*-dimensional vector of plaintext polynomials as

$$\begin{aligned} \overline{X} &= \left(\begin{array}{ccccc} \texttt{Compose} \left[\begin{array}{c} X_{1} \\ \vdots \\ X_{1} \end{array}\right] & & \cdots & & \texttt{Compose} \left[\begin{array}{c} X_{\ell} \\ \vdots \\ X_{\ell} \end{array}\right] \end{array} \,\right)\\ &= \left(X_{1} \cdots X_{\ell}\right). \end{aligned} $$

The second equality is due to the fact that Compose[*a*⋯*a*]^⊤^ is equal to the constant polynomial *a*∈*R*_*t*_ (this is not obvious, and requires one to know the explicit description of the isomorphism $R_{t}\cong \mathbb {Z}_{t}^{n}$). We can now directly take the query string bits and write them as the plaintext polynomials (constant polynomials) to form the vector $\overline {X}$. We then encrypt each of the polynomials in $\overline {X}$ to form an encrypted query vector $\overline {X}_{\text {Enc}}$.

#### Compare-add-multiply

Our first approach is particularly good for scenarios where the strings are long, and there are few batches (large *ℓ*, small *B*). We homomorphically evaluate the function

1 $$ \texttt{CAM}(\overline{\mathcal{D}}_{\text{Enc}}, \overline{X}_{\text{Enc}}) = \prod_{i=1}^{B} \sum_{j=1}^{\ell} \left(\left(\overline{\mathcal{D}}_{\text{Enc}}^{(i)}\right)_{j} - \left(\overline{X}_{\text{Enc}}\right)_{j} \right)^{2} \,,  $$

where (−)_*j*_ denotes the *j*-th component of a vector.

Due to batching, each subtraction followed by squaring in (1) compares *n* bits (one bit per one row in the original dataset $\mathcal {D}$) to the corresponding bit position in the query string *X*. Let *i* be the index of one of the *B* batches, and consider what happens in the sum for this particular *i*. If the *k*-th row within the *i*-th batch—i.e. the (*n*(*i*−1)+*k*)-th row in $\mathcal {D}$—matches the query string *X*, then the sum will have value 0. Otherwise it will have a non-zero value of at most *ℓ*. Finally, evaluating the product results in a ciphertext with a 0 in the *k*-th slot precisely when the query matched the (*n*(*i*−1)+*k*)-th row of $\mathcal {D}$ for at least one batch index *i*.

Note that we need the parameter *t* in the encryption scheme to be bigger than *ℓ*. This is to avoid false positives from appearing as a result of the sum wrapping around *t*. Note also that we do not need to care about *t* being large enough to support multiplication over the batches, because *t* being prime ensures that the product is 0 (mod *t*) precisely when one of the factors is 0.

Evaluating the function CAM requires (2*ℓ*−1)*B* additions or subtractions, *ℓ* *B* squarings, and *B*−1 multiplications. It has multiplicative depth 1+⌈log2*B*⌉, which is small when *N* is small, and most importantly does not depend on *ℓ*. It is in fact possible to evaluate CAM with only a depth ⌈log2(*B*+1)⌉ circuit, but this is computationally less efficient, and less amenable to multithreading.

##### Larger base.

If the strings to be compared are long, much of the running time of CAM will be spent performing the *ℓ* *B* squarings. The situation can be significantly improved by reducing the length *ℓ* of the rows of $\mathcal {D}$ by not representing them as strings of bits, but instead as strings of base-*b* digits, where *b*>2. Of course the same kind of representation has to be used for the query string *X*. Nearly everything presented above still works, and in particular the function CAM behaves analogously, i.e. it results in a ciphertext with a 0 in the *k*-th slot precisely when the query matches the (*n*(*i*−1)+*k*)-th row of $\mathcal {D}$ for at least one batch index *i*. The only major difference is that we must be very careful now not to wrap around *t* during the computation to avoid false positives. The sums of squares of differences in (1) can now be as large as *ℓ*_*b*_(*b*−1)^2^, where *ℓ*_*b*_ is the length of the string when represented in base *b*. For example, if the rows are *ℓ*-bit integers, we can get *ℓ*_*b*_=⌈*ℓ*/ log2*b*⌉. Once again, since *t* is a prime, we do not need to worry about the product.

To give some concrete numbers, consider bits strings of length 100. In the binary representation it would suffice to take *t*>100, but in base-16 we need *t*>5625. Roughly estimating, this increase in *t* can result in the ciphertext noise growing by 6 extra bits in each multiplication, amounting to a total of 6(1+⌈log2*B*⌉) bits more noise in the result, potentially prompting an increase in the encryption parameters when *B* is large. Nevertheless, the number of squarings is reduced by a factor of 4, so as long as the parameters do not need to be increased (at least too much), the result can be a significant improvement in performance.

##### Other considerations.

The CAM method is very efficient when the bit strings are long, and *B* is not too large. The possibility of using a larger base for encoding the strings can help further reduce their length, and increase the performance. The CAM method is also fully compatible with all performance improvements described below, making it significantly more powerful than what is suggested by the above analysis.

The downside of the CAM method is that it only reports whether the query string *X* was found in $\mathcal {D}$ or not. It is hard to see how it could be improved to support partial, or fuzzy queries, or made to return some function of the rows that were found to match the query.

#### Compare-multiply-add

Our second approach is particularly good for scenarios where the strings are short, and there are many of them (small *ℓ*, large *N*). We homomorphically evaluate the function

2 $${} \texttt{CMA}(\overline{\mathcal{D}}_{\text{Enc}}, \overline{X}_{\text{Enc}}) = \sum_{i=1}^{B} \prod_{j=1}^{\ell} \left[ 1 - \left(\left(\overline{\mathcal{D}}_{\text{Enc}}^{(i)}\right)_{j} - \left(\overline{X}_{\text{Enc}}\right)_{j} \right)^{2} \right]\!.  $$

Due to batching, each subtraction followed by squaring in (2) compares *n* bits (one bit per one row in the original dataset $\mathcal {D}$) to the corresponding bit position in the query string *X*. Since the result is subtracted from 1, a match is indicated by a resulting value of 1 rather than by a 0, as was the case in the CAM method. As the comparison results of different bits are multiplied together, a match of the entire string is indicated by a 1 (after the multiplication over the index *j*), and a mismatch by a 0. Summing over the batch index *i* results in a ciphertext with a positive entry in the *k*-th slot if the *k*-th row within the *i*-th batch for some *i*—i.e. the (*n*(*i*−1)+*k*)-th row in $\mathcal {D}$—matches the query string *X*. Otherwise the value at the *k*-th slot will be 0. Moreover, the number in the *k*-th slot will be precisely the sum of matches found in the *k*-th slot of all batches, and the sum of the values in all slots will be precisely the number of matches found.

Evaluating the function CMA requires (*ℓ*+1)*B*−1 additions or subtractions, *ℓ* *B* squarings, and (*ℓ*−1)*B* multiplications. We ignore the very cheap *plain* subtractions, where we subtract from an unencrypted number 1. It has multiplicative depth 1+⌈log2*ℓ*⌉, which is small when *ℓ* is small, but does not depend on *N*. It is possible to evaluate CMA with only a depth ⌈log2(*ℓ*+1)⌉ circuit, but this is computationally less efficient, and less amenable to multithreading.

Since the expression in the square brackets in (2) has always value either 0 or 1, the only restriction on *t* is to take *t*>*B* to prevent modular reduction in the summation phase (compare this to the bound *t*>*ℓ* in the CAM method).

##### Other considerations.

The CMA method can be very effective when *ℓ*, i.e. the length of the strings, is short. In this case the multiplicative depth does not depend on the number of rows in the dataset, which makes this method particularly suitable for situations where level 1+⌈log2*ℓ*⌉ circuits can be computed with reasonable parameters. Unfortunately, the computational complexity and the multiplicative depth quickly become very high when *ℓ* grows.

Another significant advantage of the CMA approach is that the signal of success comes in a much more useful form that in the CAM approach. For example, consider the case of only one batch. If a match is found, the result of CMA is a ciphertext with a 1 exactly in the slot(s) where the match occurred, and 0 elsewhere. Thus, the result of CMA can be used to perform conditional computations depending on whether a match was found or not. Furthermore, CMA always shows the exact number of matches that were found, which is in general not true for CAM, although it will be when we start applying hashing techniques.

Unfortunately, the CMA method does not support the *larger base* optimization to reduce the length *ℓ* of the dataset strings.

#### Compare-add

Consider the case where $\overline {\mathcal {D}}$ has only one row, i.e. only one vertical batch per string bit is needed. Let

3 $$ \texttt{CA}(\overline{\mathcal{D}}_{\text{Enc}}, \overline{X}_{\text{Enc}}) = \sum_{j=1}^{\ell} \left(\left(\overline{\mathcal{D}}_{\text{Enc}}^{(1)}\right)_{j} - \left(\overline{X}_{\text{Enc}}\right)_{j} \right)^{2} \,.  $$

The function CA has the very nice property that the *k*-th slot will end up containing the Hamming distance between the query string and the *k*-th row of the dataset. Thus, a match can be detected as a 0 in a particular slot. For this to work correctly, it is necessary to have *t*>*ℓ* to prevent modular reduction from taking place in the summation.

##### Other considerations.

This method is very fast and efficient at detecting partial matches. Unfortunately, it only works when the batch count *B* is 1.

#### Message expansion

Note that in all of the methods described above the query will have size 1/*B* of the size of the entire encrypted dataset. The result always consists of one single ciphertext, which is (1/*ℓ* *B*)-th of the size of the entire encrypted dataset. Thus, when *B* is small, the large query size can make it unreasonably inefficient to use the functions CAM, CMA, and especially CA. In some cases the assumption is that several queries will be submitted to the encrypted dataset, in which case only the amortized query size might matter. Later we discuss query packing techniques that yield significantly improved amortized performance in these cases.

### Hashing

In this section we show how a technique called *permutation-based cuckoo hashing* can be used in various ways to improve the performance of the string matching algorithms. First, it can be used to shorten the strings that need to be homomorphically compared, resulting in overall better performance in the functions CAM, CMA, and CA. Second, it allows CAM to always return the *exact* number of matches found. Third, when using CAM or CA, it allows us to pack several queries together into so-called *multiqueries*, resulting in significantly improved amortized performance in both query size and running time.

#### Permutation-based hashing

Permutation-based hashing [27] is a technique that has been used extensively to improve the efficiency of modern Private Set Intersection (PSI) protocols (see e.g. [1, 2]), where two distrusting parties both hold sets of bits strings, and want to find the intersection of their sets without revealing anything else to each other. Permutation-based hashing can be used to shorten the strings that need to be compared in these protocols, resulting in improved performance. We employ the same trick.

Let *X* be an *ℓ*-bit string, and split it into two parts as *X*=*X*_*L*_∥*X*_*R*_. Let *ℓ*_*L*_ be the bit-length of *X*_*L*_, and *ℓ*_*R*_ the bit-length of *X*_*R*_. Let $H:\{0,1\}^{\ell _{L}} \rightarrow \{0,1\}^{\ell _{R}}$ be a hash function. We define the *location* of *X* as Loc(*X*)=*H*(*X*_*L*_)⊕*X*_*R*_, where ⊕ denotes binary XOR. Consider now a hash table with *n* bins, where $\phantom {\dot {i}\!}n = 2^{\ell _{R}}$, and insert *X*_*L*_ in the bin with index Loc(*X*).

Two different strings can never yield the same value in the same bin, because Loc(*X*)=Loc(*Y*) (same bin) together with *X*_*L*_=*Y*_*L*_ (same value) imply immediately that *X*=*Y*. In some sense permutation-based cuckoo hashing encodes a part of the string into the index of the bin (location in the hash table). If we can make bin comparisons cheap (or free), the amount of work that needs to be done in actual string comparison operations may be significantly reduced. For this to work, we need each bin to contain at most one item, which depending on the hash function *H*, the total number of strings, and the hash table size *n* may or may not be likely to happen. The standard trick to make this happen is by using *cuckoo hashing*, which is a hashing technique with a particularly high load factor.

#### Permutation-based cuckoo hashing

In the above description of permutation-based hashing we assumed that each bin will end up containing at most one value. This is not hard to achieve by taking the table to be much larger than the number of possible strings to be inserted, but this is hugely wasteful. Instead, we use a hashing technique known as cuckoo hashing [28–32], which uses several hash functions to try to find a hashing scheme that gives as small of a hash table as possible, while ensuring that each bin ends up with at most one item.

##### Cuckoo hashing.

Let *n* be the size of the hash table, and suppose we have *N* items to be inserted. Let *H*_0_ and *H*_1_ be independent and random hash functions $\{0,1\}^{\ell _{L}} \rightarrow \{0,1\}^{\ell _{R}}$. We denote Loc_*i*_(*X*)=*H*_*i*_(*X*_*L*_)⊕*X*_*R*_, where *X*_*L*_ and *X*_*R*_ are as before. The *N* items are inserted into the table as follows. Given an item *X*, insert *X*_*L*_ at Loc_1_(*X*). If this location was already occupied by $X^{\prime }_{L}$ for some other item *X* ^′^ with Loc_*i*_(*X* ^′^)=Loc_1_(*X*), then pop $X^{\prime }_{L}$, and insert it at Loc_1−*i*_(*X* ^′^). Continue in this way until the conflict is resolved, and until all items are inserted. This method can fail due to an infinite loop of insertions occurring, but it is likely to succeed as long as *n*≥2*N*.

A subtle issue with the approach above was pointed out in [2, 33]. Namely, when using more than one hash function in permutation-based hashing, it is possible that two distinct items hash to the same value in the same bin, breaking the nice property that the location and the hash value uniquely describe the item. This problem can be fixed in a number of ways, e.g. by appending the index of the hash function to the string stored in the bin, or to the bin index. We choose to append it to the bin index. Thus, we take the size of the hash table to be $\phantom {\dot {i}\!}n = 2^{\ell _{R} + 1}$, and set

$$ \texttt{Loc}_{i}(X) = i\cdot 2^{\ell_{R}} + \left[ H_{i}(X_{L}) \oplus X_{R} \right] \,. $$

##### *d*-cuckoo hashing.

The space-efficiency of cuckoo hashing can be significantly improved by using more hash functions. The generalization which we call *d-cuckoo hashing* was described and analyzed in [31, 32]. Consider *d* independent and random hash functions

$$ H_{0},\ldots,H_{d-1}:\{0,1\}^{\ell_{L}} \rightarrow \{0,1\}^{\ell_{R}} \,, $$

and denote

$$ \texttt{Loc}_{i}(X) = i\cdot 2^{\ell_{R}} + \left[ H_{i}(X_{L}) \oplus X_{R} \right] \,. $$

*N* items are inserted into a hash table of size $n = 2^{\ell _{R} + \lceil \log _{2} d \rceil }$ using the random-walk method of [32] as follows. To insert an item *X*, choose a random hash function index *i*, and insert *X*_*L*_ at Loc_*i*_(*X*). If the location was already occupied by $X^{\prime }_{L}$ for some other item *X* ^′^ with Loc_*j*_(*X* ^′^)=Loc_*i*_(*X*), then pop $X^{\prime }_{L}$, randomly choose another hash function index *j* ^′^≠*j*, and insert $X^{\prime }_{L}$ at $\texttt {Loc}_{j'}(X')\phantom {\dot {i}\!}$. Continue in this way until the conflict is resolved, and until all items are inserted.

##### Success probability.

The probability that the method described above will fail due to an infinite loop increases as *N* approaches the hash table size *n*. In practice, the random walk not will not be able to find a valid configuration, where each item is mapped to a unique location. While increasing the number of hash functions *d* increases the probability of finding a valid configuration, the underlying problem of too many collisions Loc_*i*_(*X*)=Loc_*j*_(*X* ^′^) remains, unless the ratio *n*/*N* is increased.

It is important to be able to estimate the failure probability for hashing the dataset. This is because of our security model, which assumes that the dataset size is known to the adversary. If the hashed dataset is unexpectedly large (resp. small), the adversary will learn something about the dataset: it caused unusually many (resp. few) hash collisions. Thus, for the security proof to go through, it is important to determine *n* and *d* before hashing the dataset in such a way that the failure probability is at most 2^−*λ*^, where *λ* is the *statistical security parameter*. This guarantees that the adversary will not learn any extra information, except perhaps with probability at most 2^−*λ*^. Typically a *λ* of around 30 or 40 is deemed sufficient.

Several works [1, 31, 32] have analyzed the failure probability of *d*-cuckoo hashing, both from an asymptotic and a concrete perspective. While the asymptotic results can be informative, they do not give concrete parameters for desired statistical security levels. We instead follow the lead of [1], and empirically evaluate the failure probability by repeatedly constructing more than 2^30^ cuckoo tables, each with random hash functions. This allows us to observe the empirical failure probability 2^−*λ*^ for various values of *d* and *e*=*n*/*N*>1. In particular, we observe that for a fixed *n* and *d*≥3 there is a linear—or nearly linear—relationship between *e* and the statistical security parameter *λ*, and we interpolate the failure probability as a linear function to obtain parameters when *λ* is greater than 30, due to the prohibitive amount of time it would take to run *d*-cuckoo hashing significantly more than 2^30^ times.

**Fig. 1:**
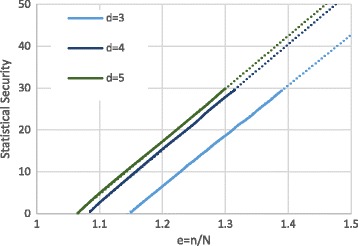
Empirical (*solid line*) and extrapolated (*dotted line*) failure probabilities of *d*-cuckoo hashing when inserting *N* items into a table of size *n*=4096. Graph describing cuckoo hashing failure probability

We provide an empirical analysis for *n*∈{4096,8192,16384}, and will see below that this is in fact sufficient to also treat huge datasets with *N*≫*n*. Figure 1 shows the empirical failure probability for *d*∈{3,4,5} and *n*=4096 for a variety of expansion factors *e*=*n*/*N*. The solid line indicates the regions where measured failure probabilities were obtained with more then 2^30^ trials, and the dotted lines denote results obtained by extrapolation. These empirical results clearly show that for *d*∈{3,4,5} the security parameter scales linearly with the expansion factor *e*.

Table 1 shows the complete set of interpolated equations, which were obtained from the empirical analysis with the parameters in question. In general, we find that the required *e* to achieve a desired security level increases as *n* becomes larger. For instance, when considering *d*=5 hash functions, *n*=4096 requires an *e* of approximately 0.01 smaller than *n*=8192, for all measured *λ*. However, this trend is weak, and may not hold for much larger *n* as the asymptotic analysis suggests. In addition, while *d*≥3 yields a linear relationship with *λ*, the special case of *d*=2 scales exponentially in the security parameter, as was also found by [1]. This is why we restrict to the much more efficient parameters of *d*≥3. Moreover, because of the diminishing returns that increasing *d* provides, we conclude that *d*=4 provides the best trade-off for the setting that we consider.

**Table 1:** The linearly interpolated lines relating the statistical security parameter *λ* to the *d*-cuckoo hash table expansion factor *e*, where *N*=*n*/*e* items are inserted to a table of size *n*∈{4096,8192,16384} using *d*∈{3,4,5} hash functions

*d*	*n*=4096	*n*=8192	*n*=16384
3	*λ*=120.5*e*−138.1	*λ*=122.5*e*−141.5	*λ*=123.3*e*−143.5
4	*λ*=126.2*e*−136.3	*λ*=128.2*e*−139.6	*λ*=127.5*e*−140.0
5	*λ*=126.1*e*−134.0	*λ*=121.3*e*−129.7	*λ*=121.5*e*−130.8

### Improved exact string matching

#### Single batch dataset

Suppose we are given a dataset $\mathcal {D}$, and that the total number of rows *N*<*n* so that the batch count *B*=1. Instead of working with $\mathcal {D}$ directly, we instead hash each row *Q* ^(1)^,…,*Q* ^(*N*)^ using permutation-based *d*-cuckoo hashing to produce a hashed dataset $H(\mathcal {D})$. The length of the rows *Q* ^(*i*)^ is *ℓ*=*ℓ*_*R*_+*ℓ*_*L*_, and $n = 2^{\ell _{R} + \lceil \log _{2} d \rceil }$. Suppose for now that *N* is so much smaller than *n* that hashing succeeds with overwhelming probability. Let $\overline {H(\mathcal {D})}$ denote the batching of the hashed dataset—analogous to $\overline {\mathcal {D}}$—and let $\overline {H(\mathcal {D})}_{\text {Enc}}$ denote its encryption.

Given a query string *X* of length *ℓ*, we need to apply permutation-based *d*-cuckoo hashing to form a hashed query. In this case we hash only one item (namely *X*) into a table of size *n*, and populate *each* of the *d* locations Loc_*i*_(*X*) with *X*_*L*_. This is necessary, because we cannot know into which of the *d* locations *X* eventually ended up when the dataset was hashed. Let *H*(*X*) denote the hash table containing the hashed query string. Each row of the hash table will have length *ℓ*_*L*_ bits, and we apply the Compose function exactly as before to form the batching of the hashed query $\overline {H(X)}$, and finally encrypt it to obtain $\overline {H(X)}_{\text {Enc}}$.

The comparison of $\overline {H(X)}_{\text {Enc}}$ with $\overline {H(\mathcal {D})}_{\text {Enc}}$ can be done using CAM, CMA, or CA, almost exactly as before, but now the strings that need to be compared have length *ℓ*_*L*_ bits, which is *ℓ*_*R*_= log2*n*−⌈log2*d*⌉ bits fewer than the original *ℓ* bits. In an optimal case *d* is as small as possible, and *n* as large as possible, but in practice *n* is bounded by performance restrictions coming from homomorphic encryption (bigger *n* means worse performance).

#### Multiple batch dataset

When the total number of items *N* is very large, it is not realistic to take *n* to be such that *N*<*n*, as this results in poor performance for homomorphic encryption. Instead, we break the dataset into several (vertical) batches, each containing at most *N* ^′^ elements, where *N* ^′^∣*N*, such that *N* ^′^<*n*. We then use *d*-cuckoo hashing to hash each of the *B*=*N*/*N* ^′^ batches of items into *B* separate hash tables of size *n*. As long *N* ^′^ is small enough, and *d* large enough, the probability of *d*-cuckoo hashing succeeding for each of the *B* hash tables is good. It is necessary to use the same set of hash functions for each of the hash tables. This technique works with both CAM and CMA.

In our security model the size *N* of the dataset and the length *ℓ* of the strings are public information. For security reasons (see below), we need to be able to fix a public predetermined choice for *B*, and say that the probability of a hashing failure occurring for at least one of these *B* batches is at most 2^−*λ*^. Building on our failure probability estimates for cuckoo hashing, it is straightforward to adjust the bounds for this setting with *B* batches: instead of solving the equations of Table 1 for a given *λ*, we must compensate for the fact that *B* such hash tables are constructed. Thus, cuckoo hashing should be expected to fail at most once in every 2^*λ*^ *B* trials. Therefore, the left hand side of the equations of Table 1 should be *λ*+ log2*B*; e.g. for *d*=3,*n*=4096 the equation becomes *λ*+ log2*B*=120.5*e*−138.1, when applied to the multiple batch setting. Note that *e*=*n*/*N* ^′^, and the total number of items in the dataset is *N*=*B* *N* ^′^=*B* *n*/*e*.

#### Larger base

In the CAM method we would still want to use larger base *b*>2 for more compact representation of the strings, and for better message expansion in encryption. The dataset and query are hashed just as before, but the items in the bins that earlier were expressed as bit strings of length *ℓ*_*L*_ are now instead expressed as base-*b* strings of length *ℓ*_*L*,*b*_=⌈*ℓ*_*L*_/ log2*b*⌉.

#### The problem of empty bins

There is one issue that we have ignored until now. The hash tables in the hashed dataset will typically contain some number of empty bins, and the hashed queries (which are also hash tables) will contain almost entirely empty bins. An empty bin naturally results in a value of 0 in the corresponding slot after batching. These zeros will cause false matches to occur, unless they are in some way invalidated.

This problem is easy to solve by writing an *impossible* value to the slots that correspond to empty bins in the batched hashed query and dataset. Note that these impossible values do also need to be different for the query, and for the dataset. Note that when using a base *b* in decomposing the strings, after batching the values in all (non-empty) slots will be at most *b*−1.

For the CAM method we populate the empty bins of the hashed dataset with the value *b*, and the empty bins of the hashed query with the value *b*+1. We conclude that the CAM method works as long as the homomorphic encryption parameter *t*>*ℓ*_*L*,*b*_(*b*+1)^2^. It is in fact possible to do slightly better by simply invalidating the unused bins for one of the *ℓ*_*L*_ positions, and require *t* to be such that (*ℓ*_*L*,*b*_−1)(*b*−1)^2^+(*b*+1)^2^<*t*.

For the CMA method the situation is a bit trickier. Suppose the batch count is *B*. Then the party that encrypts the dataset includes with it *B* additional ciphertexts that contain *masks* for the batches, invalidating (i.e. setting to zero) all locations that are empty in the hash table. Likewise, the party that submits the query includes an extra ciphertext that encrypts a mask that invalidates all locations that are empty in the query hash table. So instead of the usual CMA, we now evaluate

$$\begin{aligned} &\texttt{CMA}\left(\overline{H(\mathcal{D})}_{\text{Enc}}, \overline{H(X)}_{\text{Enc}}\right) \\&= \texttt{Mask}(H(X)) \sum_{i=1}^{B} \left\{{\vphantom{\prod_{j=1}^{\ell}}} \texttt{Mask}(H(\mathcal{D}))_{i}\right.\\ & \quad \times \!\left.\left.\left. \prod_{j=1}^{\ell} \left[{\vphantom{\left(\left(\overline{H(\mathcal{D}^{(i)})}_{\text{Enc}}\right)_{j} - \left(\overline{H(X)}_{\text{Enc}}\right)_{j} \right)^{2}}} 1 \right.\right. - \left(\left(\overline{H(\mathcal{D}^{(i)})}_{\text{Enc}}\right)_{j} - \left(\overline{H(X)}_{\text{Enc}}\right)_{j} \right)^{2} \right]\right\}\,, \end{aligned} $$

where $\texttt {Mask}(H(\mathcal {D}))_{i}$ is a batched ciphertext that has a 1 in each slot that corresponds to a non-zero hash table bin in $H(\mathcal {D}^{(i)})$, and a 0 in the rest of the slots, and Mask(*H*(*X*)) is a batched ciphertext that has a 1 in the slots that correspond to non-empty bins, and a 0 in other slots. The masks will now automatically invalidate all rows that are not supposed to be included in the comparison by setting them to zero.

### Multiqueries

Suppose the dataset has been hashed and encrypted as described above, and instead of one query *X*, consider submitting *k* queries *X* ^(1)^,…,*X* ^(*k*)^. Naively, the performance and communication cost is *k*-fold compared to submitting a single query. Alternatively, we could try to use the same hash table when hashing each of the *k* queries, cutting down the performance to (1/*k*)-th of that of the naive approach. This will work as long as for each of the *d* location functions the locations Loc(*X* ^(*j*)^) are distinct. In case there is overlap in the locations, we need to split the multiquery into two or more parts. More precisely, if *B*_*X*_ denotes the size of the largest bin after inserting all *k* items with all *d* hash functions, then we need to break the multiquery up into *B*_*X*_ hash tables, each of which will be batched and encrypted separately.

#### Success probability

We assume the number of *k* of concurrent queries (*k*-multiqueries) to be public information. For security reasons, we need to be able to predetermine a value for *B*_*X*_ that is exceeded with probability at most 2^−*λ*^, where again *λ* is the statistical security parameter. Then we always submit *B*_*X*_ separate queries, because otherwise someone observing the queries can tell whether hash collisions occurred more or less than expected, which leaks information.

Distinct location functions are constructed to map values to non-overlapping regions in the hash table. Each such region has size *n*/*d*. Thinking of balls and boxes, we need to first determine how likely it is that when placing *k* balls into *n*/*d* boxes the largest box has size at most *B*_*X*_:

$$\begin{aligned} &\operatorname{Pr}[\text{at least one box contains more than \(B_{X}\) balls}] \\ \leq &\frac{n}{d} \cdot \operatorname{Pr}[\text{first box contains more than \(B_{X}\) balls}] \\ = &\frac{n}{d} \sum_{i=B_{X} + 1}^{k} \binom{k}{i} \left(\frac{d}{n}\right)^{i} \left(1 - \frac{d}{n}\right)^{k-i} \,. \end{aligned} $$

To fail, this must occur in at least one of the *d* regions. Therefore,

$${} \begin{aligned} \operatorname{Pr}[\text{multiquery packing failure}] &\leq n \sum_{i=B_{X} + 1}^{k} \binom{k}{i}\\ &\quad\times\left(\frac{d}{n}\right)^{i} \left(1 - \frac{d}{n}\right)^{k-i} \,. \end{aligned} $$

A few examples are presented in Table 2 (for *λ*=30) and Table 3 (for *λ*=40).

**Table 2:** Parameters with multiquery failure probability ≈2^−30^

*d*=3	*d*=4				
*n*	*k*	*B*_*X*_	*n*	*k*	*B*_*X*_
4096	4	3	4096	10	4
4096	13	4	4096	27	5
4096	35	5	4096	57	6
4096	76	6	8192	4	3
8192	5	3	8192	16	4
8192	20	4	8192	45	5
8192	59	5	8192	102	6
8192	134	6

**Table 3:** Parameters with multiquery failure probability ≈2^−40^

*d*=3	*d*=4				
*n*	*k*	*B*_*X*_	*n*	*k*	*B*_*X*_
4096	5	4	4096	10	5
4096	13	5	4096	23	6
4096	30	6	8192	6	4
8192	7	4	8192	16	5
8192	21	5	8192	39	6
8192	52	6			

Furthermore, if more than one multiquery is performed over the lifetime of the data, it might be necessary to adjust the success probability to ensure that the failure probability is negligible even when performing some predetermined number *M* of *k*-multiqueries. Namely, we should select *B*_*X*_ in such a way that on expectation at most one of every 2^*λ*^ *M* *k*-multiqueries fails. For simplicity, we ignore this correction for now on, and assume *M*=1.

It is also possible to ignore this information leak, and simply submit a *k*-multiquery in as few batches as possible. The information leak resulting from this seems very difficult for an adversary to use, in particular if it does not know the hash functions used by the location functions. Nevertheless, our proof of security requires the number *B*_*X*_ to be determined beforehand, and that the multiquery size does not depend on the individual query strings themselves.

### Our protocol

In this section we describe in detail our protocol for querying an encrypted dataset using the CAM method with *d*-cuckoo hashing for multiqueries. Protocols for using CMA and CA are similar, and we omit their formal descriptions.

#### Formal protocol description

We consider two parties:

- The *server* who stores the encrypted dataset and evaluates the string matching function CAM.
- The *client* who owns the encrypts the dataset, submits encrypted queries to the server, owns the secret key, and obtains the results of the queries.

It is easy to extend our protocol to work instead with a dataset owner who is *different* from the client, and uses only the client’s public key to encrypt its dataset. Indeed, the protocol would be nearly identical, except that the server would have to randomize the non-zero slots in the output of CAM to not leak extra information about the dataset to the client. We have chosen to restrict to the two-party version of the protocol to make the security model, and the description of the protocol simpler.

**Fig. 2:**
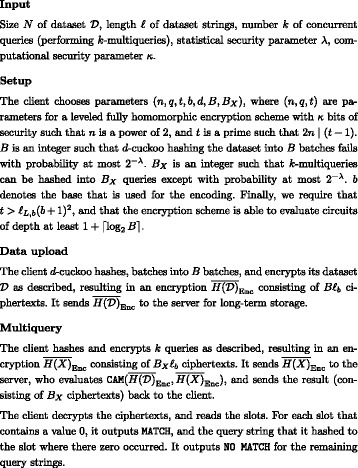
Protocol *Π*_CAM_ Protocol description

We assume that the size *N* of the dataset $\mathcal {D}$, the length *ℓ* of the strings, and the number of *k* of queries are public information. A formal description of our protocol *Π*_CAM_ is given in Fig. 2.

#### Security

Our protocol works in the semi-honest setting, where the client can have complete knowledge of the dataset and queries. As such, we need not consider whether additional information is revealed to them. The server, on the other hand, should only learn the size of the dataset, and the number of queries that are made. At the core of the security argument is a reduction to the underlying homomorphic encryption scheme, and the fact that hashing failures occur with negligible probability for the parameters used. Even though we only presented a protocol for using CAM, a similar type of protocol works with CMA and CA, and the proof of security does not depend in a significant way on which string matching method is used.

First, let us consider the setup phase where the encrypted dataset is constructed. When constructing the hashed dataset $H(\mathcal {D})$, failing to construct the *B* cuckoo hash tables obtained within it may reveal to the server that a certain number of hash collisions occurred while hashing the dataset. As such, we must ensure that the probability of this event is negligible in the statistical security parameter *λ*. Our hashing failure probability estimates show how the public parameters *n*,*d*, and *B* can be chosen to have this property. Such a choice is made in protocol *Π*_CAM_ (Fig. 2). Once the hashed dataset $H(\mathcal {D})$ is constructed, it is encrypted with homomorphic encryption as was described above, the exact details depending on whether CAM, CMA, or CA is used. The encrypted dataset is then uploaded to the server.

Later, a query of size *k* is made. The exact details depend slightly on whether CAM, CMA, or CA is used. However, in all cases the query is encrypted using homomorphic encryption. Moreover, the size of the query is a function of the public parameters *k*,*N*,*ℓ*, and *b*. A full simulation of the protocol can therefore be obtained by replacing $\mathcal {D}$ and the query with uniformly sampled ones of the same size. This change is computationally indistinguishable by a straightforward reduction to the indistinguishability property of the homomorphic encryption scheme.

## Results

In this section we will give an example application that was one of the problem tracks in the iDASH Secure Genome Analysis Competition in 2016. Track number 3 in the competition—*Testing for Genetic Diseases on Encrypted Genomes*—can be described as follows:

**Problem description.** Consider a setup with a client (or possibly several clients), and a server. Initially the client holds genomic data in VCF files in plaintext form. It encrypts the files using homomorphic encryption, and uploads them to the server for long-term storage. At a later point the client wishes to query the server for the presence of a particular entry (line) in the encrypted data. The query will also be encrypted using homomorphic encryption to prevent the server from learning any of the client’s private data from it. More generally, the client can issue multiqueries, in which several entries are queries at once. After the client gets an encrypted result back from the server, it decrypts and decodes it to learn a Boolean result: MATCH or NO MATCH (or *k* Boolean results for a *k*-multiquery).

We consider two scenarios. In the first one we assume that the VCF files contain up to 10^4^ rows, and in the second one up to 10^5^ rows. We present separate parameters optimized for both scenarios.

**Parsing the files.** The client starts with parsing the VCF files as follows. It reads in a line, and outputs #CHROM (chromosome number), POS (position), the last entry in the string REF (one of {*A*,*C*,*G*,*T*,empty}), the last entry in the string ALT (similarly one of {*A*,*C*,*G*,*T*,empty}), and one bit representing whether SVTYPE=SNP. We encode the symbols in REF and ALT using numbers as

$$ \{A=0,\,C=1,\,G=2,\,T=3,\,\text{empty}=0\}\,. $$

The empty symbol appears for example when SVTYPE=DEL. Substitutions (SVTYPE=SUB) and insertions (SVTYPE=INS) are ignored to obtain better performance, and because the competition problem description allowed it. This is why we only care about the last (and only) symbol in the REF and ALT strings. While our implementation only supports querying entries with SVTYPE=SNP, but it is possible—and easy—to generalize our solution to support more general queries at the price of only slightly reduced performance. This is because the CAM function behaves very nicely with increasing string length.

After each row has been represented as integers in this way, we convert it to binary representation. Since #CHROM is an integer between 0 and 22, or a symbol *X* or *Y*, we need 5 bits to represent it. Since POS is an integer of length at most 9 decimal digits, we need 30 bits to represent it. Since we only encrypt homomorphically the last symbol of the REF and ALT strings, we need 2 bits for both. Finally we need one bit to represent whether SVTYPE=SNP. In total, our encoding of each row has length *ℓ*=40 bits. For each row we will encrypt homomorphically only these 40 bits.

Thus, we encode an entry of the VCF file into 40 bits (to be homomorphically encrypted) as follows:

$$\begin{array}{*{20}l} x &= \underbrace{x_{1} \ldots x_{5}}_{\text{Chromosome number}} \| \underbrace{x_{6} \ldots x_{35}}_{\text{Starting position}}\\ &\quad\| \underbrace{x_{36} x_{37}}_{\text{Reference}} \| \underbrace{x_{38} x_{39}}_{\text{Alternative}} \| \underbrace{x_{40}}_{\text{Is SNP? }} \end{array} $$

**Encrypting the dataset.** In order to reduce the bit length of the inputs, we use permutation-based 4-cuckoo hashing. We use *n*=8192, and *N* ^′^=7700. We consider two types of situations, where in the first one *N*=10^4^ (so *B*=2), and in the second one *N*=10^5^ (so *B*=13). Now, *d*=4, so *ℓ*_*R*_= log2*n*−⌈log2*d*⌉=11, and *ℓ*_*L*_=*ℓ*−*ℓ*_*R*_=29. The location functions are Loc_*i*_(*x*)=*i*·2^11^+[*H*_*i*_(*x*_*L*_)⊕*x*_*R*_] for *i*∈{0,…,3}. With table size this much larger than the number of items inserted in each table, 4 hash functions suffices to obtain a good probability of succeeding in the hashing process. Each data entry (also each query) is represented by 40 bits, which hashing reduces to *ℓ*_*L*_=29 bits. We use a block size *b*=2^10^ to get *ℓ*_*L*,*b*_=3. Thus, we need *t*>*ℓ*_*L*,*b*_(*b*+1)^2^=3151875. In our first example with *N*=10^4^ and *B*=2 this results in an encrypted dataset consisting of *B* *ℓ*_*L*,*b*_=6 ciphertexts. Similarly, in our second example with *N*=10^5^ and *B*=13 we obtain an encrypted dataset consisting of *B* *ℓ*_*L*,*b*_=39 ciphertexts.

For the sake of performance, in our example we ignore the possibility of a (very) minor information leak due to a small but non-negligible hash failure probability. Considering the case of *B*=13, to instead obtain a statistical security level of *λ*=30, it suffices to use the same parameters but to restrict the VCF files with at most 78781 rows (*N* ^′^≈6060). To obtain a statistical security level of *λ*=40, the VCF files should have no more than 74483 rows (*N* ^′^≈5729).

**Encrypting multiqueries.** We restricted to multiqueries of size at most 5 due to the specifications of the competition problem description. Again, we ignored potential information leaks from hash failures, in all examples producing only a single set of *ℓ*_*L*,*b*_ ciphertexts (*B*_*X*_=1). Indeed, for *n*=2^13^, and *d*=4, packing a 5-multiquery into one succeeds with probability approximately 0.98. Thus, in our implementation, the 5-multiqueries consist of only *B*_*X*_ *ℓ*_*L*,*b*_=3 ciphertexts. The amortized size of each query is therefore (3/5)/39=1/65 of the size of the encrypted dataset.

Even if a hash collision would occur, and the multiquery would have to be split up, it is very hard to see how an adversary could use this information, since typically it would not even know what the hash functions are. Nevertheless, to instead obtain a statistical security level of *λ*=40 would require taking *B*_*X*_=4. Alternatively, *B*_*X*_=4 allows for up to 16-multiqueries while still achieving a statistical security level of *λ*=30.

### Implementation and performance

For homomorphic encryption we use the Simple Encrypted Arithmetic Library - SEAL [4], which implements the Fan-Vercauteren encryption scheme [3]. Table 4 summarizes the SEAL encryption parameters that we used. In fact, we used two sets of parameters: one for our smaller example (*N*=10^4^), and another one for the larger one (*N*=10^5^). Note that both parameter sets use the same *n* (exponent in poly_modulus), but the smaller parameters have a much smaller *q* (coeff_modulus). The larger parameters are estimated to have a computational security level *κ*≈100 bits [34], and the smaller parameters have a significantly *higher* security level, which we did not explicitly estimate. The larger parameter set can easily be upgraded to support over 128 bits of security if needed, and this will come at a slight decrease in performance.

**Table 4:** SEAL encryption parameters for *N*=10^4^ (left) and *N*=10^5^ (right)

SEAL parameter	*N*=10^4^	*N*=10^5^
poly_modulus	*x* ^8192^+1	*x* ^8192^+1
coeff_modulus	2^155^−2^25^+1	2^253^−2^21^ +5·2^14^+1
plain_modulus	3686401	3686401
decomposition_bit_count	78	52

**Table 5:** 5-multiquery performance results with *N*=10^4^ (left) and *N*=10^5^ (right)

	*N*=10^4^	*N*=10^5^
Operation	Time (ms)	
Encoding dataset	115	1098
Encrypting dataset	120	956
Encoding 5-multiquery	9	9
Encrypting 5-multiquery	60	75
Evaluating CAM	225	2003
Decrypting response	15	19
Data description	Size (KB)	
Original VCF	557	5490
Parsed VCF	196	1920
Encrypted dataset	2305	19971
Encrypted 5-multiquery (*B*_*X*_=1)	1158	1545
Encrypted response (*B*_*X*_=1)	386	515

The results of our experiments are presented in Table 5. The experiments were ran on an Intel Xeon E5-1620 v3 @ 3.50 GHz, with 16 GB of RAM installed. Our implementation used 8 threads to speed up the evaluation of CAM. For homomorphic encryption we used SEAL v2.1.

## Discussion

Our results in Table 5 demonstrate that homomorphic encryption can be practical for outsourcing data storage, and enabling privacy-preserving queries on the encrypted data with realistic performance. Message expansion due to homomorphic encryption is not prohibitively big (10–12x based on Table 5), and we certainly expect the situation to improve during the next few years as a result of new innovations in cryptography and encoding techniques. Computational performance is already surprisingly good, and could easily be improved further by using a homomorphic encryption library that has a stronger emphasis on performance optimizations, such as FV-NFLlib [35]. We were only concerned about simple set membership queries in this work, and it remains a challenge to create efficient protocols that allow more flexible types of queries.

We demonstrated our techniques in the context of querying SNPs in encrypted (parsed) VCF files of up to 10^5^ rows. By increasing the homomorphic encryption parameters it becomes possible to query also much larger files (millions of rows).

Our protocol relies heavily on the semi-honest security model, which is a standard assumption to make due to the often huge performance benefits it yields over protocols secure under stronger security models, where the parties behavior is not necessarily restricted to follow the protocol description.

## Conclusions

We have demonstrated that homomorphic encryption is indeed powerful and efficient enough to be used for storing and privately querying encrypted genomic data for the presence of mutations. We also showed that the computational and communication cost can be dramatically reduced in the amortized setting, where the presence of several mutations is queried at once.

Extending our protocol to more complicated types of queries remains an interesting and important challenge. Another possible direction for future research is to extend this work to the malicious security model, or perhaps some slightly weaker model, but doing this without accruing too much performance or communication overhead seems challenging.
